# *CKM* Gene *rs8111989* Polymorphism and Power Athlete Status

**DOI:** 10.3390/genes12101499

**Published:** 2021-09-25

**Authors:** Valentina Ginevičienė, Audronė Jakaitienė, Algirdas Utkus, Elliott C. R. Hall, Ekaterina A. Semenova, Liliya B. Andryushchenko, Andrey K. Larin, Ethan Moreland, Edward V. Generozov, Ildus I. Ahmetov

**Affiliations:** 1 Department of Human and Medical Genetics, Institute of Biomedical Science, Faculty of Medicine, Vilnius University, 01513 Vilnius, Lithuania; valentina.gineviciene@mf.vu.lt (V.G.); audrone.jakaitiene@mf.vu.lt (A.J.); algirdas.utkus@mf.vu.lt (A.U.); 2 Research Institute for Sport and Exercise Sciences, Liverpool John Moores University, Liverpool L3 5AF, UK; elliotthall@live.co.uk (E.C.R.H.); E.Moreland@2017.ljmu.ac.uk (E.M.); 3 Department of Molecular Biology and Genetics, Federal Research and Clinical Center of Physical-Chemical Medicine of Federal Medical Biological Agency, 119435 Moscow, Russia; alecsekaterina@gmail.com (E.A.S.); zelaz@yandex.ru (A.K.L.); generozov@gmail.com (E.V.G.); 4 Research Institute of Physical Culture and Sport, Volga Region State University of Physical Culture, Sport and Tourism, 420010 Kazan, Russia; 5 Department of Physical Education, Plekhanov Russian University of Economics, 115093 Moscow, Russia; andryushenko-lil@mail.ru; 6 Laboratory of Molecular Genetics, Kazan State Medical University, 420012 Kazan, Russia

**Keywords:** anaerobic performance, creatine kinase, genetic variant, genotype

## Abstract

Multiple genetic variants are known to influence athletic performance. These include polymorphisms of the muscle-specific creatine kinase (*CKM*) gene, which have been associated with endurance and/or power phenotypes. However, independent replication is required to support those findings. The aim of the present study was to determine whether the *CKM* (*rs8111989*, c.*800A>G) polymorphism is associated with power athlete status in professional Russian and Lithuanian competitors. Genomic DNA was collected from 693 national and international standard athletes from Russia (*n* = 458) and Lithuania (*n* = 235), and 500 healthy non-athlete subjects from Russia (*n* = 291) and Lithuania (*n* = 209). Genotyping for the *CKM* rs8111989 (A/G) polymorphism was performed using PCR or micro-array analysis. Genotype and allele frequencies were compared between all athletes and non-athletes, and between non-athletes and athletes, segregated according to population and sporting discipline (from anaerobic-type events). No statistically significant differences in genotype or allele frequencies were observed between non-athletes and power athletes (strength-, sprint- and speed/strength-oriented) athletes. The present study reports the non-association of the *CKM* rs8111989 with elite status in athletes from sports in which anaerobic energy pathways determine success.

## 1. Introduction

Human physical performance is genetically influenced, particularly in sprint/power sports, with traits relating to muscular strength and power tending to have greater heritable components than endurance phenotypes [1]. Although the genetic contribution to athletic performance is undoubted, a conclusive list of the genetic variants involved, and the mechanisms underpinning their effect, remain unknown. Among several genes previously associated with athletic performance, the role of the muscle-specific creatine kinase (*CKM*) in cellular energy homeostasis has led to the emergence of the *CKM* gene as an important candidate [2,3].

Muscular energy provision is maintained by adenosine triphosphate (ATP) breakdown and the concomitant release of free energy. To sustain contractile activity, the rate of ATP regeneration must be complementary to ATP demand and is facilitated by the phosphagen, glycolytic, and mitochondrial respiration energy systems. Each system has specific substrates and byproducts, in addition to differences in maximal rates and capacities for ATP regeneration [4]. During short-duration, maximal-effort activities such as sprinting, strength training, and high-power sports, the circulatory system cannot fully supply the exercising musculature with the oxygen required for adequate mitochondrial respiration. Consequently, the phosphagen system utilizes intramuscular stores of high-energy phosphates, with creatine phosphate (CrP) being the primary component of this system. The resynthesis of ATP from adenosine diphosphate (ADP) is catalyzed by the enzyme creatine kinase (CrP + ADP + H+ ⇌ ATP + Cr), meaning the efficiency of ATP generation is affected by the activity of creatine kinase [4,5].

Creatine kinase (CK) can be comprised of the M (muscle) and B (brain) subunits, assembled into the homodimeric CK-M and CK-B enzymes or the CK-MB heterodimer. Each is expressed in a tissue-specific manner, with CK-M being the most abundant in skeletal muscle and CK-MB activity being the greatest in cardiac muscle. The CK-B homodimer is primarily expressed in nervous tissue, with small concentrations in skeletal muscle, whereas the additional CK isoforms Scmit-CK and Umit-CK are localized to the mitochondria. The abundance of CK-M in skeletal muscle means it is the most important CK isoform for energy regulation during exercise, with recent evidence suggesting that the CK-PCr system activity is genetically influenced [5]. The CK-M-encoding *CKM* gene is mapped to chromosome 19q13.2, and various studies have addressed the gene’s role in skeletal muscle energy metabolism. A seminal study by van Deursen and colleagues showed that CK-M-knockout mice were unable to sustain maximal isometric contractions but had greater fatigue resistance and could perform a higher number of contractions to exhaustion [6]. Subsequent studies in rat models demonstrated that both the duration and intensity of endurance training have a negative linear relationship with *CKM* expression [7]. Additional animal studies focused on the consequences of *CKM* knockout in different muscle fiber types, reporting that *CKM* activity is inversely correlated with aerobic capacity and endurance performance. Accordingly, targeted deletion using animal models has permitted the greater understanding of the role of CK-M in exercise performance, suggesting that higher expression may be detrimental to aerobic capacity but advantageous during anaerobic activity.

Human studies have focused on sequence variations in coding and noncoding regions of the *CKM* gene. More than 260 *CKM* single-nucleotide polymorphisms (SNPs) have been identified [8]. The most studied *CKM* SNP is rs8111989, where adenosine (A) is substituted with guanine (G) at nucleotide position 800 (c.*800A>G) [8]. Despite being located in the 3′ untranslated region and without altering the amino acid composition of CK-M, this variant purportedly affects *CKM* gene expression and CK-M activity [9]. Since the association of the rs8111989 A allele with endurance phenotypes before and after aerobic training was reported by Rivera and colleagues [10], several studies of the rs8111989 SNP have been published [3,5,11,12,13,14,15]. However, there is still a lack of consensus regarding the influence of the rs8111989 A and G alleles on athletic ability, meaning that replication studies using independent populations are needed to confirm or reject the association of rs8111989 with performance phenotypes. Therefore, the aim of this study was to determine whether the *CKM* (A/G) rs8111989 polymorphism is associated with elite power status in a cohort of Lithuanian and Russian athletes. We hypothesized that rs8111989 genotype distribution would differ between elite power athletes and non-athletes, and that the frequency of the G allele and GG genotype would be higher in athletes competing in sports that are reliant on anaerobic energy production.

## 2. Materials and Methods

### 2.1. Subjects and Ethical Approval

All procedures in this study conformed with ethical standards concerning the scientific research of sport and exercise, and were approved by the local Ethics Committees (Federal Research and Clinical Center of Physical-Chemical Medicine and the Lithuanian Bioethics Committee). Written informed consent was obtained from all participants and the study was conducted in compliance with the Declaration of Helsinki.

This case-control study included 458 Russian (RU) and 235 Lithuanian (LT) professional athletes (431 males and 262 females, aged 25.0 ± 6.5 years). All athletes were ranked in the top 10 nationally for their respective sporting disciplines and were prospectively stratified into three groups according to the duration and distance of their event, spanning a spectrum from sprint-oriented (athletes from strength/power sports with some aerobic component) to strength-oriented (athletes from strength/power sports with a predominantly anaerobic component). The sprint-oriented group (RU: n = 171; LT: n = 83) included athletes competing in short-distance races, including 100–400 m track-and-field sprinters, sprint cycling, rowing/canoe, and canoe-kayak sprints, 50–ß100 m swim and 500–1000 m track races. The strength-oriented group (RU: n = 105; LT: n = 29) included weightlifters and powerlifters. The speed-/strength-oriented group (RU: n = 182; LT: n = 123) included athletes from track-and-field (throwing (push-throw), jump events (high jump, long jump, triple jump, and pole vault)), figure skating, alpine skiing, pentathlon, decathlon, heptathlon, rock climbing, rhythmic gymnastics, and wrestling. The inclusion criteria stipulated that athletes must have no history of positive tests for performance-enhancing substances under standard anti-doping controls. Non-athlete participants were 500 healthy, unrelated citizens (347 males and 153 females, aged 29.0 ± 8.5 years) from Russia (*n* = 291) and Lithuania (*n* = 209), all of whom had no competitive sporting experience. All participants were Caucasians.

### 2.2. Genotyping

For all Lithuanian samples, genomic DNA was extracted from peripheral blood leukocytes of the Lithuanian participants using a standard phenol-chloroform extraction method. Genotyping for *CKM* rs8111989 was performed in duplicate using an allelic discrimination assay on a 7900HT Fast real-time polymerase chain reaction (PCR) instrument (Applied Biosystems™, Life Technologies, Waltham, MA, USA) with TaqMan probes (TaqMan^®^ Pre-Designed SNP Genotyping Assay ID:C_3145002_10; Applied Biosystems™, Life Technologies, Waltham, MA, USA). Genotypes were assigned using software (SDS Software v2.3, Applied Biosystems^TM^). In order to ensure proper internal control, positive and negative controls from different DNA aliquots were used for each genotype analysis.

For all Russian samples, molecular genetic analysis was performed with DNA samples obtained from leukocytes (venous blood). Four milliliters of venous blood was collected in tubes containing EDTA (Vacuette EDTA tubes; Greiner Bio-One, Kremsmünster, Austria). Blood samples were transported to the laboratory at 4 °C, and DNA was extracted on the same day. DNA extraction and purification were performed using a commercial kit (Techno-Sorb, Moscow, Russia) according to the manufacturer’s instructions (Technoclon, Moscow, Russia), which included chemical lysis, selective DNA binding on silica spin columns, and ethanol washing. The extracted DNA quality was assessed by means of agarose gel electrophoresis. The genotyping process was performed using HumanOmni1-Quad BeadChips or HumanOmniExpress BeadChips (Illumina, San Diego, CA, USA) to genotype > 900,000 SNPs, including *CKM* rs8111989. The assay required 200 ng of DNA sample as input with a concentration of at least 50 ng/µL. Exact concentrations of DNA in each sample were measured using a Qubit Fluorometer (Invitrogen, Waltham, MA, USA). All further procedures were performed according to the instructions of the Infinium High-Density Assay. Ten percent of samples were genotyped twice with a 100% success rate of reproducibility.

### 2.3. Statistical Analyses

Genotype frequencies of athletes and non-athletes were tested for compatibility with the Hardy–Weinberg equilibrium (HWE). Differences in genotype and allele frequency were compared using the Chi-squared goodness-of-fit test. The homogeneity hypothesis for genotype and allele frequency differences between groups were assessed by Chi-squared or Fisher’s exact tests where appropriate. Odds ratios (OR) with 95% confidence intervals (CIs) were calculated in cases where there was a significant difference in the genotype distribution between groups. The level of significance was set at *p* < 0.05. All analysis was performed using the SPSS statistical software package (IBM SPSS v.21).

## 3. Results

The distributions of *CKM* rs8111989 genotypes in athletes versus controls are presented in Table 1. Genotype distributions were in the Hardy–Weinberg Equilibrium for athletes and non-athletes (in all groups tested separately, *p* > 0.05).

No statistically significant differences in *CKM* genotype or allele distribution were observed compared to non-athletes when all athletes were combined, nor when athletes were segregated by country (Table 1). In addition, *CKM* genotype and allele frequencies did not differ between male and female athletes and non-athletes for each nationality.

## 4. Discussion

Sports events that involve short intense efforts are often categorized as anaerobic athletic activities. Each of these events may emphasize different anaerobic phenotypes, for example, sprinting relies mostly on speed, weightlifting or powerlifting rely mainly on muscle strength, and throwing and jump events rely on the combination of speed and strength [15,16,17]. Therefore, the aim of this study was to investigate differences in the *CKM* rs8111989 genotype between three groups of elite athletes from anaerobic-type events: sprint-oriented, strength-oriented, and speed/strength-oriented athletes. We chose to study the rs8111989 genetic variant because the *CKM* gene has emerged as a potential candidate to affect the development of anaerobic phenotypes. We hypothesized that a genetic predisposition for high CK-M protein activity in skeletal muscle may be favorable to power/strength athletes, and specifically that the GG genotype would be more common in strength and power competitors, due to previous associations with increased CK-M activity. This theory is supported by rodent models documenting the inverse relationship of *CKM* activity with endurance capacity [7], suggesting that high *CKM* activity may impair aerobic performance yet favor anaerobic activity. This study led us to conclude that the genotype and/or allele frequencies of the *CKM* rs8111989 polymorphism do not seem to influence performance in either the total strength/power group or in sports groups from two discrete populations. Nevertheless, this study emphasizes the importance of considering well-defined groups of athletes with a clear phenotype in genetic association studies applied to elite sport performance.

Muscular strength is a determinant of general athletic ability in many sports, particularly in short-distance sprinting, and strength and power events, in which the physiological characteristics favorable to national and international standard competition are well documented [12,15,16,18]. In contrast with endurance competitors, strength and power athletes typically exhibit greater percentages of fast-twitch muscle fibers than slow-twitch fibers [17], and those fast-twitch fibers are suited to the shorter, more powerful muscle contractions reliant on anaerobic energy pathways. The muscle-specific CK-M enzyme catalyzes the reversible transfer of energy-rich phosphate from CrP to ADP and is essential for energy provision during contractile activity, with higher CK-M activity in fast-twitch compared to slow-twitch fibers [4,5]. Consequently, the *CKM* gene has emerged as a candidate that might affect the development of aerobic and/or anaerobic phenotypes. Furthermore, according to the GTEx portal, the G allele of the rs8111989 polymorphism is associated with decreased expression of the *MARK4* gene in skeletal muscle, which acts as a negative regulator of the mTORC1 complex (a key factor in maintaining skeletal muscle mass) [19]. Therefore, we hypothesized that a genetic predisposition for high CK-M protein activity may be advantageous to athletes in sports that are reliant on the contractile capacity of fast-twitch muscle fibers, and specifically that variants associated with increased CK-M and mTORC1 activity would be more common in strength competitors. The A-to-G substitution of the rs8111989 SNP does not change the CK-M amino acid sequence but reportedly alters *CKM* mRNA stability [12] and potentially CK-M expression. Further studies are required to determine whether this mechanism influences *CKM* expression and skeletal muscle phenotypes.

Our findings are not supported by a recent meta-analysis of *CKM* rs8111989 genotypes and/or allele distribution in athletes and non-athletes, where the frequencies of the G allele and GG genotype were higher in power athletes than in non-athletes [8]. The rs8111989 SNP may also contribute to baseline aerobic capacity and differences in the response of maximal oxygen uptake to endurance training [10,11,20,21]. Fedotovskaya and colleagues reported that the AA genotype is associated with high values of maximal oxygen uptake [14], whereas the G allele has been associated with combat athlete status in Polish and Russian populations [22]. Although also reliant on fast-twitch muscle fibers, sprint- and speed/strength-oriented sports involve a greater aerobic contribution than weightlifting and powerlifting, potentially explaining why the GG genotype was not overrepresented in these cohorts. Athletes with the rs8111989 AA genotype are also six times more likely to suffer exertional muscle breakdown than AG or GG genotypes [11,23], leading to the suggestion that the G allele protects against exertional muscle damage [13,24]. Since fast-twitch fibers are more susceptible to exertional muscle damage [24], genetic resistance to exertional muscle damage would be advantageous to strength-oriented athletes who perform repetitive maximal contractions. However, the findings of the present study do not indicate that the rs8111989 G allele carriers are predisposed to excel in strength and power sports.

A growing body of evidence supports an association of the rs8111989 AA and GG genotypes with endurance and strength phenotypes, respectively [12,17,21,22,25,26,27,28]. However, a tendency for G allele carriers to achieve higher maximum oxygen uptake values has been reported [27], and several studies (together with the present study) have failed to detect any association with athlete status or performance [8,9,10,13,23]. This demonstrates that further investigation surrounding *CKM* variants and athletic performance phenotypes is required. To date, 62 genetic markers have been associated with strength-power athlete status [3], but only some were reported in strength sports groups (weightlifting and powerlifting). These include SNPs of *CNTFR, ACTN3, AGT, AR,* *HIF1A, PPARA, PPARG, PPARGC1A*, and other genes [3,9,17,29,30,31,32,33,34,35,36]. Recently, Grishina and colleagues confirmed the association of three SNPs (rs12055409, rs4626333, and rs2273555) with strength measures in weightlifters, which were supported by functional evidence [31,32], whereas the most comprehensive study of elite strength athletes (weightlifters and powerlifters) to date documented 28 SNPs associated with athlete status [33]. Of those 28 SNPs, the *LRPPRC* rs10186876, *MMS22L* rs9320823, and *PHACTR1* rs6905419 polymorphisms were also associated with competitive weightlifting performance. Importantly, that study was the first to demonstrate the likelihood that achieving elite status in strength sports depends on a high number of SNPs [33]. Therefore, further studies are needed to replicate those findings and to confirm whether those SNPs are associated with performance in strength-oriented sports. This study does not confirm the previous associations of the *CKM* rs8111989 GG genotype with power athlete status [22]. Additional studies of elite power athletes from different nationalities are required to further substantiate our findings.

We acknowledge the limitations of the present study. Firstly, we did not include endurance athletes and are unable to ascertain whether the rs8111989 A and G alleles are associated with contrasting performance phenotypes. Second, participant nationalities were restricted to Russia and Lithuania, meaning that the associations described in the present study cannot be generalized to athletes from other countries or geographical ancestries. We also did not collect direct measures of athletes’ strength and power. However, our inclusion criteria stipulated that all athletes had competed at the national or international level, which would not be possible without the physiological characteristics associated with elite strength phenotypes. Lastly, we studied a singular SNP and recognize that genetic association studies represent only the first steps toward understanding the genetic factors influencing performance traits. However, candidate gene association studies are crucial to the direction of new analyses, such as those incorporating modern DNA technologies and bioinformatics, to further analyze the heritability of physical capacity and to work toward the application of genomic research into practice.

## 5. Conclusions

The present study reports the non-association of the *CKM* rs8111989 polymorphism with elite status in athletes from sports in which anaerobic energy pathways determine success. Therefore, the *CKM* rs8111989 polymorphism may not be considered as a candidate gene to influence the performance of power athletes from Russia and Lithuania.

## Figures and Tables

**Table 1:** Genotype and allele frequencies of the *CKM* rs8111989 SNP in athletes and non-athletes from Russia and Lithuania.

Groups	N	Allele Frequency (%)		*p*-Value vs. Non-Athletes	*CKM* Genotype Frequency (%)			*p*-Value vs. Non-Athletes
		A	G		AA	AG	GG	
All strength-oriented	134	67.2	32.8	0.496	65 (48.5)	50 (37.3)	19 (14.2)	0.246
All sprint-oriented	254	64.4	35.6	0.914	107 (42.1)	113 (44.5)	34 (13.4)	0.980
All speed/strength	305	65.2	34.8	0.720	136 (44.6)	126 (41.3)	43 (14.1)	0.556
Russian power athletes	458	65.6	34.4	0.988	204 (44.5)	193 (42.2)	61 (13.3)	0.915
Lithuanian power athletes	235	64.7	35.3	0.858	104 (44.3)	96 (40.8)	35 (14.9)	0.534
All power athletes	693	65.3	34.7	0.98	308 (44.4)	289 (41.7)	96 (13.9)	0.475
Russian non-athletes	291	66.7	33.3	-	133 (45.7)	122 (41.9)	36 (12.4)	-
Lithuanian non-athletes	209	60.3	39.7	-	74 (35.4)	104 (49.8)	31 (14.8)	-
All non-athletes	500	64.0	36.0	-	207 (41.4)	226 (45.2)	67 (13.4)	-

## Data Availability

The data presented in this study are available on request from the corresponding authors.
